# Peer mentorship to improve outcomes in patients on hemodialysis (PEER-HD): a randomized controlled trial protocol

**DOI:** 10.1186/s12882-022-02701-1

**Published:** 2022-03-05

**Authors:** Ladan Golestaneh, Michal Melamed, Ryung S. Kim, Jennifer St. Clair Russell, Michele Heisler, Lisandra Villalba, Taylor Perry, Kerri L. Cavanaugh

**Affiliations:** 1 Department of Medicine, Division of Nephrology, Albert Einstein College of Medicine/ Montefiore Medical Center, Bronx, NY 10467 USA; 2 Department of Epidemiology and Population Health, Albert Einstein College of Medicine, Bronx, NY 10461 USA; 3 Department of Medicine, Division of General Internal Medicine, Duke University, Durham, NC 27701 USA; 4 Department of Medicine, Institute for Healthcare Policy and Innovation, University of Michigan, Ann Arbor, MI 48109 USA; 5 Department of Nephrology and Hypertension, Vanderbilt University Medical Center, Nashville, TN 37232 USA; 6 Vanderbilt Center for Effective Health Communication, Vanderbilt University Medical Center, Nashville, TN 37232 USA

**Keywords:** Hemodialysis patients, Peer mentorship, Hospitalization, Self-management, Self-efficacy, Clinical trial

## Abstract

**Background:**

Patients receiving in-center hemodialysis experience disproportionate morbidity and incur high healthcare-related costs. Much of this cost stems from potentially avoidable hospitalizations. Peer mentorship has been used effectively to improve outcomes for patients with complex chronic diseases. We propose testing the efficacy of peer mentorship on hospitalization rates among patients receiving hemodialysis.

**Methods:**

This is a multicenter parallel group randomized controlled pragmatic trial of patients treated at hemodialysis facilities in Bronx, NY and Nashville, TN. The study has two phases. Phase 1 will enroll and train 16 hemodialysis patients (10 in Bronx, NY and 6 in Nashville TN) to be mentors using a program focused on enhancing self-efficacy, dialysis self-management and autonomy-supportive communication skills. Phase 2 will enroll 200 high risk adults receiving hemodialysis (140 in Bronx, NY and 60 in Nashville, TN), half of whom will be randomized to intervention and half to usual care. Intervention participants are assigned to weekly telephone calls with trained mentors (see Phase 1) for a 3-month period.

The primary outcome of Phase 1 will be engagement of mentors with training and change in knowledge scores and autonomy skills from pre- to post-training. The primary outcome of Phase 2 will be the composite count of ED visits and hospitalizations at the end of study follow-up in patient participants assigned to intervention as compared to those assigned to usual care. Secondary outcomes for Phase 2 include the change over the trial period in validated survey scores measuring perception of social support and self-efficacy, and dialysis adherence metrics, among intervention participants as compared to usual care participants.

**Discussion:**

The PEER-HD study will test the feasibility and efficacy of a pragmatic peer-mentorship program designed for patients receiving hemodialysis on ED visit and hospitalization rates. If effective, peer-mentorship holds promise as a scalable patient-centered intervention to decrease hospital resource utilization, and by extension morbidity and cost, for patients receiving maintenance in-center hemodialysis.

**Trial registration:**

Clinicaltrials.gov identifier: NCT03595748; 7/23/2018.

**Trial sponsor:**

National Institutes of Diabetes, Digestive and Kidney Disease (NIDDK) 5R18DK118471.

**Funding:**

Funding for this study was provided by the National Institutes of Diabetes, Digestive and Kidney Disease: R18DK118471.

**Study status:**

This is an ongoing study and not complete. We are still collecting data for observational follow-up on participants.

**Related articles:**

No related articles for this study have been submitted to any journal.

The study sponsor and funders had no role in the design, analysis or interpretation of this data. The content is solely the responsibility of the authors and does not necessarily represent the official views of the National Institutes of Health.

**Supplementary Information:**

The online version contains supplementary material available at 10.1186/s12882-022-02701-1.

## Background

The high rate of hospitalization among patients receiving maintenance hemodialysis (MHD) contributes to enormous cost, morbidity and decreased quality of life [1–3]. Variability in hospitalization rates across communities in the United States suggests modifiable risk factors for avoidable hospitalizations [1, 4]. MHD specific patient knowledge, effective self-management behaviors and easy access to quality ambulatory healthcare have been identified as modifiable factors [5–7]. Barriers to adoption of effective self-management behaviors in patients receiving MHD include: 1)poor understanding of the rationale and metrics of estimated dry weight and the link between urea clearance, nutrition and uremic symptoms; and 2) under-utilization of healthcare resources available in the outpatient setting [8–11]. Low self-efficacy, frequently linked to poor social support, and its association with underutilization of available resources and non-adherence to hemodialysis treatments also contribute to hospitalization risk [5, 6]. Patient education materials provided by dialysis organizations do not increase patient self-efficacy or engagement with self-management as these documents contain complex medical jargon and provide only general guidelines, not patient specific instructions [12, 13].A multi-component program including novel, scalable strategies that can provide needed social support to improve patients’ self-efficacy and self-management is critically needed.

Peer mentorship has been used to improve self-management behaviors and outcomes in chronic diseases and could be effective in counteracting modifiable mediators of avoidable hospitalization among patients receiving MHD [14]. Peer mentors are individuals who have overcome similar challenges with whom patients can identify and trust. Peer mentorship improved adherence and satisfaction with care in a study of patients on MHD, but the study lacked the power and duration to show improvements in hard clinical outcomes, such as hospitalizations and mortality [15]. Although a number of patient advocacy organizations, including the end stage renal disease (ESRD) National Coordinating Center, the Midwest Kidney Network and the National Kidney Foundation (NKF) have utilized various peer mentorship approaches for patients receiving MHD, to date no studies have examined the effect of this type of intervention on clinical outcomes including hospitalization [15–19].

The overarching goal of this research study is to test the effect on the composite rate of ED visits and hospitalizations of a peer mentor intervention for patients receiving MHD.

## Methods

The purpose of this manuscript is to present the study design and protocol, and adaptations implemented after the initial COVID-19 surge, of the PEER-HD trial using the SPIRIT framework, a standardize approach to describing protocols [20].

### Participants, interventions and outcomes

#### Trial design

PEER-HD is a parallel group, randomized, controlled, multi-center pragmatic trial that tests the superiority, as compared to usual care, to precent hospitalizations of a telephone delivered, protocol-guided, mentoring intervention among patient receiving MHD.

#### Study setting

**Fig. 1:**
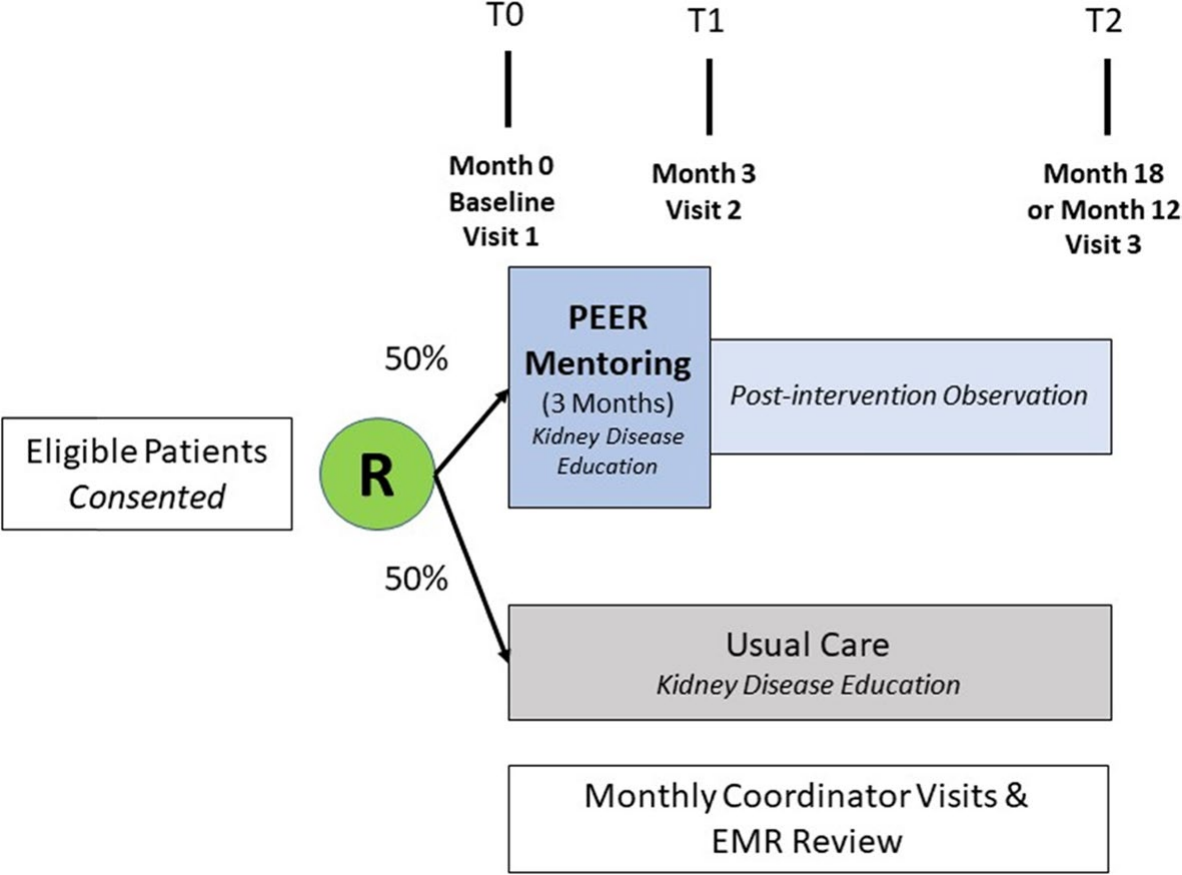
Trial Design-Phase 2 with Patient Participants

The study will enroll patients receiving MHD from two geographic locations; the first sample will be from eight dialysis facilities in the Bronx, NY where Einstein/ Montefiore faculty are medical directors and the second sample will be from two Vanderbilt affiliated dialysis facilities in Nashville, TN (Fig. 1).

#### Eligibility criteria

Mentor participants and patient participants must meet designated inclusion and exclusion criteria that differ for each group to be considered for recruitment (Table 1).

**Table 1:** Eligibility for Participants (inclusion and exclusion criteria)

**Mentors**	
**Inclusion Criteria**	**Exclusion Criteria**
Age > 21 years	Cognitive or psychiatric illness precluding participation as determined by dialysis physician
1) Receives in-center hemodialysis for ESRD for at least 1 year at one of the participating dialysis facilities; 2) No hospitalizations in previous 6 months;,and 3) No unexcused absence or shortening of dialysis treatments in the past 6 months; and 4) use of AVF/G access, and 5) serum albumin > 3.5 mg/dLin the month prior to enrollment	Less than a 6-month life expectancy
Willing to give informed consent for 24 months of training and intervention delivery	Active substance use, excluding cannabinoids
Speak Spanish or English	Enrolled in another Peer Support or Educational Study
**Patient Participants**	
**Inclusion Criteria**	**Exclusion Criteria**
Age > 21 years	Cognitive or psychiatric illness precluding participation as determined by dialysis physician
1) One or more hospitalizations or ED visits in the previous month or 2) > 1 unexcused, missed treatment or 2 shortened dialysis treatments (by greater than 10 mins) in the last month, or 3) use of catheter as only access or 4) > 4% IDWG per week at least once in the last month or 5) serum albumin less than 3.5 mg/dL in the last month or 6) initiated dialysis in past 90 days	Less than a 6-month life expectancy
Will to give informed consent to 1) randomization and 2) telephone intervention	Active substance use, excluding cannabinoids
Speak Spanish or English	Enrolled in another Peer Support or Educational Study

### Interventions

#### Mentor participants

##### Phase 1-Mentor participants

**Fig. 2:**
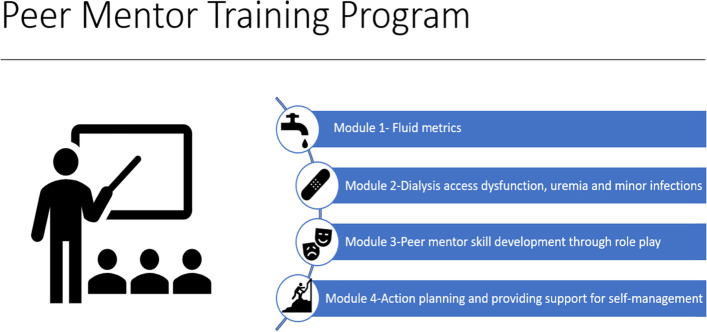
Training Program for Mentor Participants

The investigators identify and enroll 16 patients to serve as mentors (10 in the Bronx and 6 in Nashville). These mentors are trained using a semi-structured curriculum based on Peer Mentorship curricula developed by Drs. Michele Heisler and Jennifer St Clair Russell and modeled on the “information, motivation, behavior (IMB) model” [21]. Training takes place at a central location or via teleconference and is conducted over a series of 4, 2-h sessions (Supplementary Table 1 and Fig. 2). All mentors are followed for study endpoints over a total period of 12–18 months from time of enrollment. They will have a total of 3 assessments, including baseline, month 9 and end of study (at 12 or 18 months) (see below under “COVID-19 adaptations”).

##### Mentor training

The peer-mentor training manual was designed and written by the study PIs with consultant input. We employed a multidisciplinary team to finalize the training curriculum including 2 dialysis facility social workers, 2 nurse managers and 2 patient representatives (current patients on MHD who serve as ESRD network patient advocates). The curriculum aims to: a) increase knowledge about estimated dry weight (EDW), inter-dialytic weight gain (IDWG) and symptoms of fluid overload; b) increase knowledge about adherence, dialysis clearance, their relationship to each other, to symptoms of uremia and to various outcomes; c) to improve dialysis self-management by identifying a dialysis specific skill set; and d) to increase peer mentor communication skills with a focus on confidentiality, motivational interviewing, non-judgmental listening, social modeling and providing social support (Supplementary Table S1; Fig. 2) [22–24].

##### Phase 2-trial period for Mentor participants

Mentors receive a study-provided mobile telephone that is used for all intervention calls. The trial period begins after all mentors are trained and mentors are assigned up to 3–5 patient participants (Fig. 1). Each mentor makes weekly calls to assigned patient participants over the duration of 3 months. At the end of 3 months that mentor is assigned a group of different patient participants. The flexible number of participants assigned to mentors at any one time is to accommodate variability in patient-participant enrollment success over the course of the study period. At each weekly call the mentors discuss topics related to hemodialysis or other issues that are brought forth by assigned intervention patient participants. As much as possible, mentors record the content and impressions of each conversation in notebooks provided by staff. The content structure of telephone calls is flexible to accommodate the needs of individual patient participants. The study staff check on mentors on a regular basis to address challenges the mentors are facing, provide brief booster support and ensure they can continue performing their duties (see Supplementary materials).

##### Mentor participant compensation

All mentors are compensated $50 for each of 3 primary study visit assessments. Mentors will be compensated $20 for each attended training session (2 h each; total: $80), for travel and for up to $15/h of time spent on the telephone while mentoring. Any telephone call lasting between 3 min and 30 min will be treated as ½ hour and any call over thirty minutes to 1 h will be treated as a full hour, for the purpose of calculating telephone intervention compensation. The study will pay up to $150/week.

#### Patient participants

##### Phase 2-trial period for patient participants

All patient participants undergo an initial assessment immediately after enrollment, an assessment at 3 months (end of intervention period) and a last assessment at the end of observational follow-up (at 12 or 18 months after enrollment) (Fig. 1). Those participants assigned to intervention receive educational materials, a study mobile telephone and weekly calls from mentors over a period of 3 months. They are asked to record their impressions of the mentoring session after each successful telephone call. Intervention patient participants also receive binders with relevant didactic materials and literature available on dialysis care including: 1-action planning (where mentors help set up and follow up on weekly self-management action plans), 2- “Kidney School” modules on how best to follow the dialysis treatment plan and 3-educational handouts developed to inform participants on dry weight metrics, hemodialysis access (NKF) and signs of uremia and infection. Participants assigned to usual care will similarly receive a packet which includes the “Kidney School” documents and educational handouts listed above.

##### Patient participant compensation

All enrolled patient participants are compensated $50 for each of 3 study visit assessments. Only those randomized to intervention receive weekly mentor telephone calls for a period of 3 months of intervention for which they are financially compensated. They receive up to $15 per hour for time spent on the telephone talking to their mentor. Any call lasting between 3 min and 30 min will be treated as ½ hour and any call over thirty minutes to 1 h will be treated as a full hour, for purposes of calculating compensation. The study will pay up to 2 h of telephone time per week (up to $30/week).

#### Mentor-intervention patient participant matching

Patient participants randomized to “intervention” are assigned to mentors using the following criteria: language (English or Spanish), age (within one decade), sex, and race/ethnicity. Every effort is made to match by these criteria. However, because of the limited number of mentor participants priority is given to language ability as the matching criteria. If a patient assigned to intervention becomes hospitalized during the 3-month intervention period, the mentors is instructed to continue calls as per their schedule where feasible.

### COVID-19 adaptations (starting from month 9 from initiation of study activities until the end of the study period)

COVID-19 related adaptations were made to the protocol as soon as research study activities were allowed to resume after the initial pandemic surge in New York City (March–June 2020). All three PIs and the study team members were involved in making Data Safety and Monitoring Board (DSMB) and IRB approved adaptations involving the nature of study coordinator interactions with participants (for assessments) and truncation of observational follow-up period. Because of the disruption in trial activities and to meet initial enrollment targets, the DSMB committee recommended to truncate observational follow up from 15 months to 9 months (and total follow up time from 18 months to 12 months) in all of those participants (mentors and patient participants) with < 12 months of follow-up at the end of April 2021. COVID-19 disrupted all in-person processes; thus, all enrollment and training activities were conducted virtually (using Zoom) and the study protocol and participant consents were amended to reflect these changes. Because the intervention is delivered by telephone, disruptions to study processes introduced by COVID-19 only affected enrollment and study-visit activities, which required conversion from in-person to virtual visits for some encounters until safety protocols to mitigate risk where available.

### Outcomes

#### Primary outcome

The primary outcome of the randomized controlled trial (RCT) is the composite rate of hospitalizations and ED visits in intervention, as compared to usual care, patient participants.

#### Secondary outcomes

Secondary outcomes include change in knowledge, self-efficacy, perception of social support, coping skills and dialysis adherence metrics gleaned from validated survey tools that were delivered before and after the intervention (see below under “Data collection methods”), in the intervention as compared to the usual care group.

Secondary outcomes for mentor participants included ascertainment of the feasibility, efficacy and fidelity of a peer-mentor training program. In addition, changes over the study enrollment period in above parameters using validated survey tools (outlined above for patient participants) will also be ascertained for mentor participants.

### Participant timeline

The study timeline is outlined in Fig. 1. Mentor enrollments and training occurs prior to patient participant enrollment. Intervention periods between mentor/patient participant pair groups is staggered based on date of patient participant recruitments and availability of mentor participants.

### Sample size

For sample size calculation for evaluation of the primary endpoint, estimates are made based on dialysis facility hospitalization events during 12 months prior to trial start at the primary study enrollment sites. For the usual care group, we expect 0.27 events per month (100 prevalent patients and 27 composite events per month). With 73 patients per group (after 25% drop-out), our study has > 80% power to detect 20% reduction (i.e. a reduction of 0.054 visits per person-month) at type 1 error level 0.05 [15]. Recruitment target for patient participants is 100 per group over the course of the study to account for potential loss to follow-up [25].

### Participant recruitment

After all required regulatory waivers are executed, potential mentors undergo a screening of their electronic health record (EHR) for eligibility criteria. Once deemed eligible, the study staff approach, consent and schedule initial assessments for potential mentor participants. Similar procedures are followed for screening and recruitment of potential patient participants. Eligibility criteria are shown in Table 1.

### Assignment of interventions

#### Allocation sequence, concealment and implementation

Enrolled patient participants are randomized after completion of informed consent and the baseline study visit, utilizing a computer-generated randomization sequence stratified by center. As each participant is enrolled, sequential envelopes with the random assignment to either the intervention or the usual care control group are given by the coordinators (1:1).

### Data collection, management, and analysis

#### Data collection methods

##### Mentor participant training assessments

Mentors will be evaluated by *post-training* assessments. These will include brief surveys assessing the knowledge of training subjects including: fluid (6 items), uremia and infection (6 items), mentor role (5 items). Each of these is scored as correct or incorrect and reported as a percentage correct (0–100%). Each mentor’s confidence with mentor skills is assessed with an 11-item survey with Likert-type responses. Each mentor also provides ratings (11-items) and open-ended comments about their satisfaction and experience with the training sessions.

##### Study assessments: baseline visit, 3-month (patient participants), 9-month (mentor), 12 or 18-month (all)

- Data regarding outcomes is collected monthly using both participant report and health records. The data is self-reported by patients during monthly phone calls made by the study coordinator. Monthly dialysis facility health records and hospital records is reviewed for each patient participant to ascertain the outcome. Dialysis adherence metrics including number of missed monthly minutes of dialysis and mean monthly IDWG are collected monthly from dialysis facility EHR.
- Survey tools will be used during study visits (*n* = 3) to assess various measures and include: 1- Chronic Hemodialysis Knowledge Survey (CHeKs) [26] questionnaire with additional dialysis-specific new knowledge items (CHeKs PLUS), self-management behaviors (Kidney Disease Behavioral Index(KDBI)) [27], self-efficacy (Perceived Kidney Dialysis Self-management Scale (PKDSMS) [27]), coping behaviors (brief COPE-kidney) [28] and Multi-dimensional Scale of Perceived Social Support (MSPSS and dialysis specific social support) [29] questionnaires. Questionnaires addressing depression (CESD-R 10) [30], communication ability (Communication Assessment Tool(CAT)) [31] and healthcare quality of life (SF36 health survey)(Rand) [32], healthcare environment (Health Care Climate Questionnaire (HCCQ) Long Form) [33] are also completed.

##### Intervention fidelity tools

Telephone billing records for all mentor and intervention patient participant phones will be reviewed. All calls will be abstracted including phone number called, date, time, and duration. A summary of weekly minutes between mentor and patient participant pairs will be generated for characterization of intervention dosing as well as evaluation of execution of mentor and patient participant responsibilities. Where possible, random recordings of calls will be submitted to the study team for qualitative review of content. Call logs and self-reported phone calls with duration by mentors and mentees will also be reviewed, coded and characterized for content notes.

### Data management

All data will be recorded in a study REDCap database, with access granted to each enrollment site.

### Statistical methods

The rates of the composite event for the primary endpoint (ED visits and hospitalizations) will be determined as event per patient-month and compared between the intervention and usual care patient-participant groups using Poisson regression with two random intercepts. In the regression model, the composite counts of ED visits and hospitalization for 12- or 18-months follow-up is the outcome variable and the intervention assignment of the patient participants is the primary binary predictor. The log of follow-up time will be included as offset. The random intercepts will account for the correlation between intervention patients who share a common mentor as well as the correlation between patient participants who are from a common dialysis facility (out of 10 total facilities). We will control for type 1 error at 0.05. To test the effect of a peer mentor led telephone intervention on patient self-management skills and perceptions of support (secondary endpoints) including 1) dialysis adherence (mean number of dialysis missed minutes per month), 2) mean monthly IDWG, 3) monthly albumin level, 4) dialysis knowledge, 5) self-efficacy and 6) social support scores; we will compare each of these outcomes between the two randomization groups using linear regression with two random intercepts [34]. In the regression model, the mean dialysis missed minutes per month, mean IDWG and mean albumin level for each patient is the outcome variable and the intervention assignment of the mentees is the primary binary predictor. The random intercepts will account for the correlation between intervention patients who share a common mentor as well as the correlation between patients who are from a common facility. For the perception of social support and self-efficacy we will use linear regression with two random intercepts to compare the scores on the PKDSMS, dialysis specific self-efficacy (PDialSMS) and MSPSS between the intervention and usual care patient participants at the various time points. The outcome variable for the regression is the assessment scores and the predictor of interest is the binary indicator of intervention groups. Two random intercepts will account for the correlation between intervention patients who share a common mentor as well as the correlation between patients who are from a common dialysis facility.

We will use the Wilcoxon rank sum test to test the difference in pre- and post-training knowledge assessments in the mentors (Baseline CHeKS and CHeKS-PLUS vs. mid-study and end-of-study visits). The outcome variable for the regression is the difference between pre-and post-training scores and the parameter of interest is the intercept. For qualitative data, we will review the content of the individual interview responses and describe the content including common themes identified.

### Monitoring

#### Data safety management board (DSMB)

A board of 3 independent physicians from AECOM meet biannually with study investigators to review the study protocol. Study activities, study conduct, adverse events and serious adverse events. The investigators have the option of calling ad-hoc DSMB meetings in case of any event potentially attributable to the study intervention.

## Discussion

The PEER-HD program will test the efficacy of a telephone delivered peer mentorship on ED visit and hospitalization rates in patients receiving maintenance hemodialysis. Using a training curriculum developed by the study PIs to improve self-management skills specific to hemodialysis and communication skills (Fig. 2), mentor participants who are also patients receiving in-center hemodialysis, will be trained. Patient participants at high risk for ED visits and hospitalizations will then be identified and randomized to peer mentor intervention or usual care.

Hospitalizations drive up to 40% of the cost for dialysis care [35–37]. Patients receiving hemodialysis are hospitalized, on average, 2 times per year and over 35% of these patients are re-hospitalized within 30 days of discharge [3, 38]. Cardiovascular disease is the most common cause of hospitalizations followed by infections and vascular access complications [39–42]. Some hospitalizations may be avoidable if patients could self-monitor their interdialytic fluid intake and their vascular access. Other times patients could reach out to their outpatient clinics for help with certain symptoms, before needing to present to the ED. Those patients who skip at least one or more dialysis sessions per month, or shorten at least one treatment per month, have a significantly higher risk for increased short-term, fluid related, hospitalizations and a 25% higher risk for mortality [5, 43]. In a study of patients in a national database, up to 34% of patients skipped dialysis treatments [44]. Complications related to low dialysis adherence contribute to avoidable hospitalizations in other ways and include infection risk, uremia related malnutrition and cognitive decline [45–49]. Patient level barriers to adherence include 1) lack of knowledge, 2) negative attitudes about self-management and 3) low self-efficacy. Education provided by dialysis facilities provide general guidelines on diet and adherence but do not help patients recognize or attribute the symptoms of uremia or IDWG to poor self-management behaviors [50–58]. The use of contextually sensitive interventions that improve social support and self-efficacy improve IDWG and dialysis treatment adherence [59, 60]. Peer mentorship has been effective in chronic diseases such as diabetes mellitus, depression, AIDS, and also in patients on dialysis [14, 23, 61–67]. Peer based interventions improve health related behaviors and self-management [22, 63]. Peer mentor interventions in patients on hemodialysis have shown success in improved goals of care discussions, treatment adherence and quality of life parameters [19, 60, 68].

Limitations of our study design deserve mention. The patient population that this study targets represents the highest utilizers of hospitalization services because of various health burdens and social challenges. These qualities make them difficult to recruit in research studies resulting in risk for selection bias and under-representation of the population of most interest. However, this multi-site collaboration increases the potential diversity of the study population and its subsequent generalizability. Cognitive and health literacy challenges may result in lack of effect of the intervention. The study will, however, measure and include these potential confounders to reduce the risk of their impact on interpretation of the findings. Finally, variables not considered in the study design (such as the COVID-19 pandemic) may contribute to attrition and lack of fidelity to training and intervention schedules. Adaptations of the study protocol are instructive for future studies experiencing similar unexpected scenarios.

In conclusion, the PEER-HD pragmatic randomized clinical trial promises to shed light on the feasibility of a mentor training program, and the efficacy of a telephone delivered peer mentorship intervention on hospitalization and emergency room visit rates in patients receiving MHD. Advances in understanding this approach to deliver information, emotional and logistical support is required to inform future expansion of peer initiatives in dialysis care.

## Supplementary Information

**Additional file 1.**

## Data Availability

Study materials are available upon request. As data collection is not complete the full database has not been deposited in a public repository.

## Abbreviations

AECOM: Albert Einstein College of Medicine
VUMC: Vanderbilt University Medical Center
ESRD: End Stage Renal Disease
ED: Emergency Department
MHD: Maintenance Hemodialysis
NKF: National Kidney Foundation
IMB: Information Motivation Behavior
IDWG: Interdialytic weight gain
RCT: Randomized Controlled Trial
CHeKs: Chronic Hemodialysis Knowledge Survey
CHeKs PLUS: Chronic Hemodialysis Specific Survey
KDBI: Kidney Disease Behavioral Index
PKDSMS: Perceived Kidney Disease Self-management Scale
COPE: Brief coping behaviors questionnaire
MSPSS: Multi-dimensional Scale of Perceived Social Support
CESD-R 10: Depression screening questionnaire
CAT: Communication Assessment Tool
SF36 health survey: Healthcare quality of life
HCCQ: Healthcare climate questionnaire
PDialSMS: Perceived Dialysis Self-management Scale
DSMB: Data and Safety Management Board
