# Supplementary material for: Peer mentorship to improve outcomes in patients on hemodialysis (PEER-HD): a randomized controlled trial protocol
Source: BMC Nephrol. 2022 Mar 5;23:92. doi: 10.1186/s12882-022-02701-1 (PMC8897762; doi:10.1186/s12882-022-02701-1)
Supplement: Supplementary file 1 — Additional file 1. [file 12882_2022_2701_MOESM1_ESM.docx]

**SUPPLEMENTARY MATERIALS**

Training program details

The training program will include 4 sessions with the first two (a and b) using didactic lectures for increasing knowledge and test knowledge after the training with structured surveys (Supplementary Table S1). We will focus on elucidating patient level factors for accurate EDW determination and achievement of EDW, as well as successful access self-management (Supplementary Table 2(. These factors will likely include increased understanding of rationale and metrics of fluid management and dialysis adequacy, their relation to dialysis adherence and to vascular access, how to advocate for more hours of dialysis to enable EDW achievement and adequate dialysis as well as to maintain a healthy diet and how to recognize the symptoms of fluid overload or uremia. There will be an emphasis on how to communicate with the facility staff and nephrologists with regards to fluid related symptoms such as shortness of breath, swelling, fatigue and severe cramping or post dialysis fatigue and uremic symptoms such as pruritis, dysgeusia, and nausea (Supplementary Tables 1 and 2). With relation to vascular access knowledge, we will clarify signs of vascular access infection such as fever and pain, and clotting such as a decrease or cessation of thrill. We will also educate patients with catheters on the dangers of ongoing catheter use. (Supplementary Table S2)

For parts c and d of the training course we will focus on teaching the following skills to our mentors: a) confidentiality and setting boundaries with intervention patient participants, b) motivational interviewing, c) non-judgmental listening, d) emotional support, and e) assistance with tailored goal setting and problem solving, f) increasing perception of self-efficacy and g) modeling self-management behaviors.(Supplementary Tables 1 and 2) This part of the curriculum will be semi-structured, informed by the Information-Motivation-Behavior (IMB) framework, and employ group discussions by mentors, role playing and mentee simulations that will operationalize concepts related to effective mentoring skills.

Ensuring intervention fidelity by mentor participants.

To ensure fidelity of the mentors to the training and to their responsibilities during the trial period Study staff will do monthly “call-ins” with mentor participants and will use the “Study Staff Monthly Mentor Check-in” survey. This survey is designed to probe mentors on whether they feel overwhelmed or dissatisfied with the study responsibilities. Study staff will also check in with mentor participants after each intervention period (every 3 months) and fill the “Assessment of Mentors Performance and Quarterly Intervals”. This assessment will allow mentors to rate themselves on their performance as mentors. A remediation plan will allow the study staff to contact mentor participants in cases where intervention patient-participants or study staff determine that mentors are unable to perform the duties allotted to them. This remediation plan is aimed at mentor participants who are identified as underperforming.

Supplementary Table S1: Mentor Training Curriculum Overview

| **Mentor training curriculum**  **4 session, 2 hours each schedule** | **Educational material to be used** | **Tools to assess baseline and follow up knowledge** |
| --- | --- | --- |
| Parts a and b:  *Knowledge:*  The metrics and logic of EDW prescription and symptoms related to fluid deplete or overload states.  The metrics and logic of URR and symptoms related to uremia and how to improve dietary adherence and communication skills  How to communicate your symptoms and concerns to your care team. | *Didactic*  Educational materials from NKF. Research team to develop a list of practical questions about EDW, and fluid and dietary management  *Framework*:  Chronic Disease Self-Management Plan geared to ESRD. Input from patients and social workers. | Health literacy quiz and assessments to test EDW and IDWG calculation, and how to address low URR, low albumin with staff.  A quiz with case presentations of various concrete scenarios demonstrating effective communication about symptoms and self-management. |
| Part c and d:  *Peer mentor skills:*  Confidentiality, motivational interviewing, non-judgmental listening to provide emotional support and affirmation of emotions, social-support and self-efficacy promotion |  | Role playing, simulation, didactics of motivational interviewing. Group discussions.  Formative and summative assessments |

Supplementary Table S2: Details of Each Training Module

| **Content of Module 1** | **Content of Module 2** | **Content of Module 3** | **Content of Module 4** |
| --- | --- | --- | --- |
| **Knowledge of dialysis fluid management**   - understanding metrics of estimated dry weight (EDW) - control of fluid gains between treatments - adherence to dialysis prescribed time   **Symptom inventory**   - symptoms of volume overload - symptoms of volume depletion   **Concrete Skills**   - How to self-manage volume overload and volume depletion - Communicating with dialysis care team (staff & charge nurse) and kidney doctor - Scheduling extra dialysis - Discussing EDW prescription | **Knowledge of dialysis adequacy & time**   - Understanding uremia - Understanding symptoms of uremia   **Knowledge of dialysis access**   - Vascular access knowledge & care   **Knowledge of minor infections**   - Understanding minor infections   **Concrete Skills**   - Communication with dialysis staff and kidney doctor - Access self-monitoring - Communication with dialysis staff about minor infection concerns - Engaging urgent or primary care for evaluation of medical concerns | **Knowledge of the role and actions of a peer mentor**   - What it means to be a peer mentor in the program - Role of self-efficacy & confidence in dialysis - Understanding confidentiality - Understanding roles and boundaries for mentors   **Concrete skills**   - Communication with your mentees - Increasing mentee self-efficacy & confidence; dealing with negativity & anger - Procedures to ensure privacy & confidentiality | **Knowledge about the reasons for and methods of motivational approache**s   - Understanding motivational interviewing rationale   **Knowledge about the reasons for and methods of nonjudgmental Listening**   - Understanding the role of listening to help your mentee feel supported - Role of self-efficacy & confidence in dialysis   **Knowledge about Action Planning**   - How to discuss an action plan with your mentee - How to utilize the action plan tools   **Concrete Skills**   - Non-judgmental listening techniques - Motivational interviewing procedures - How to complete the SMART steps for a dialysis related action plan. |
